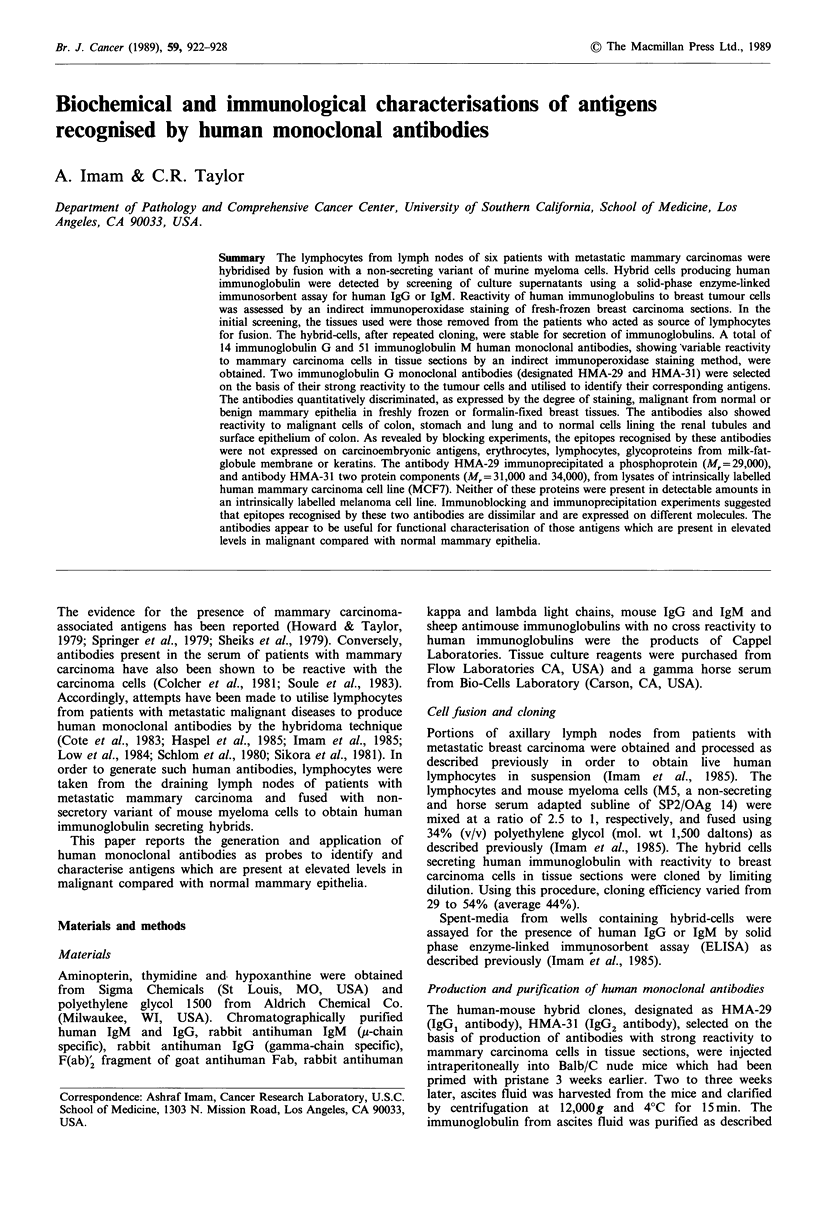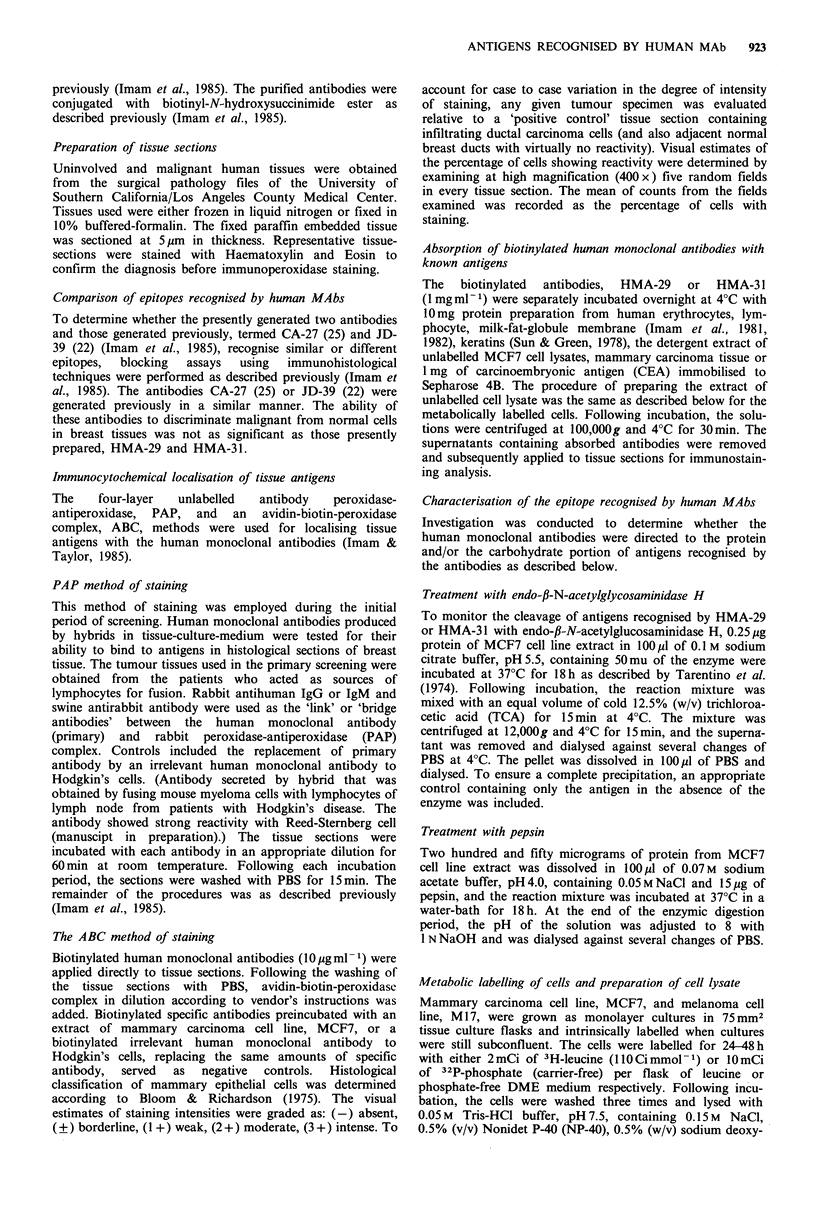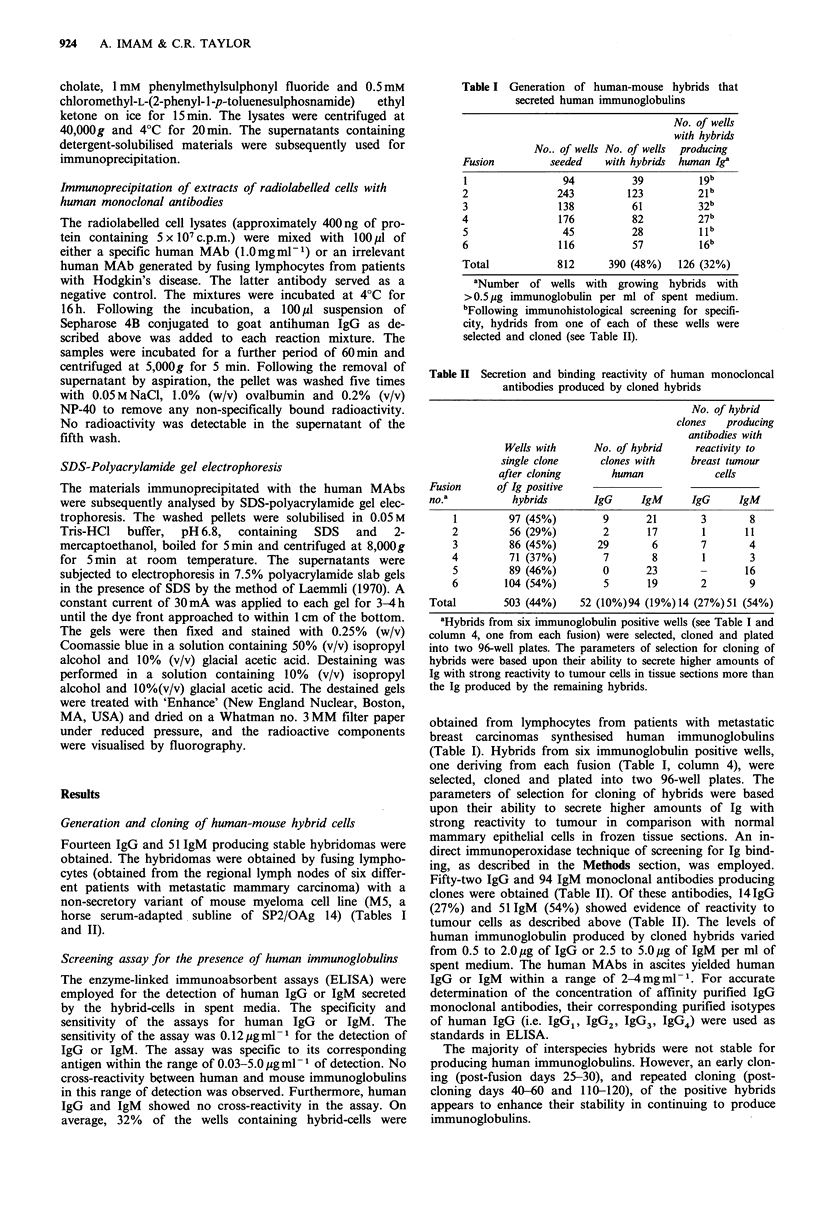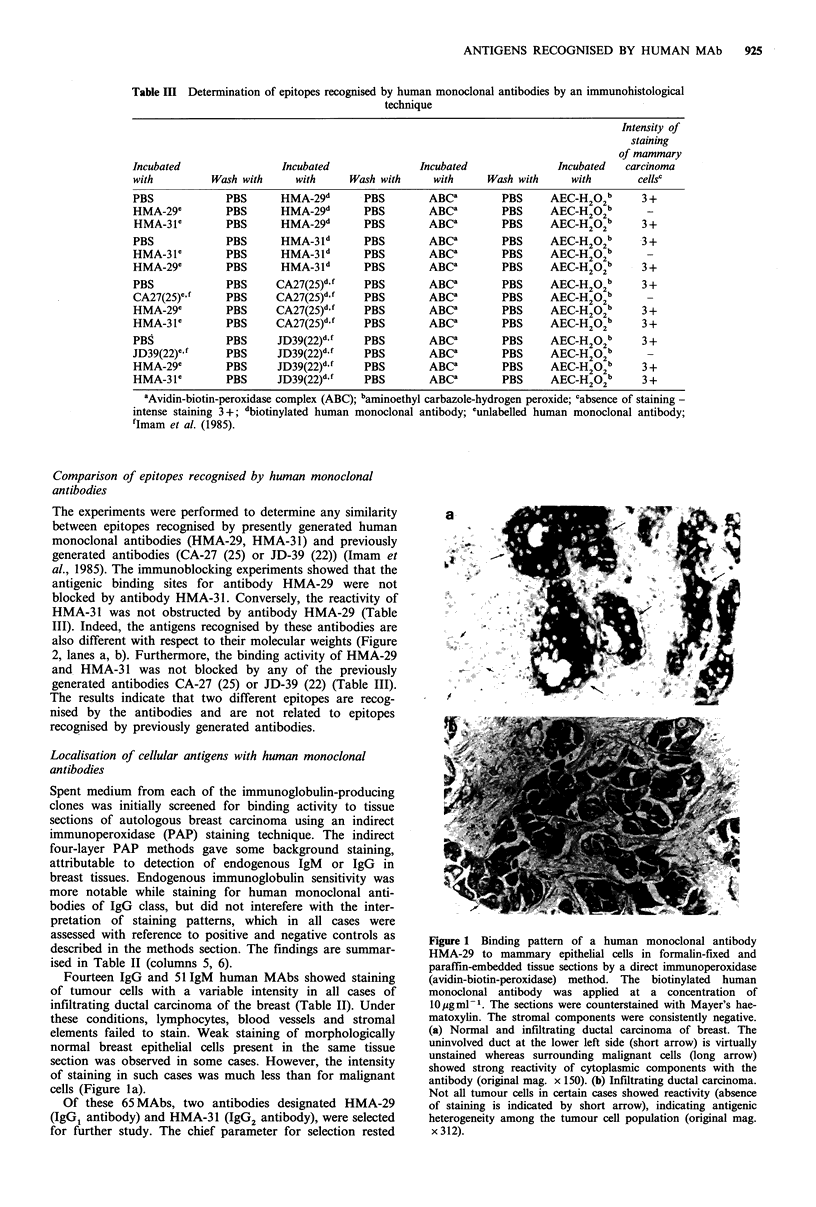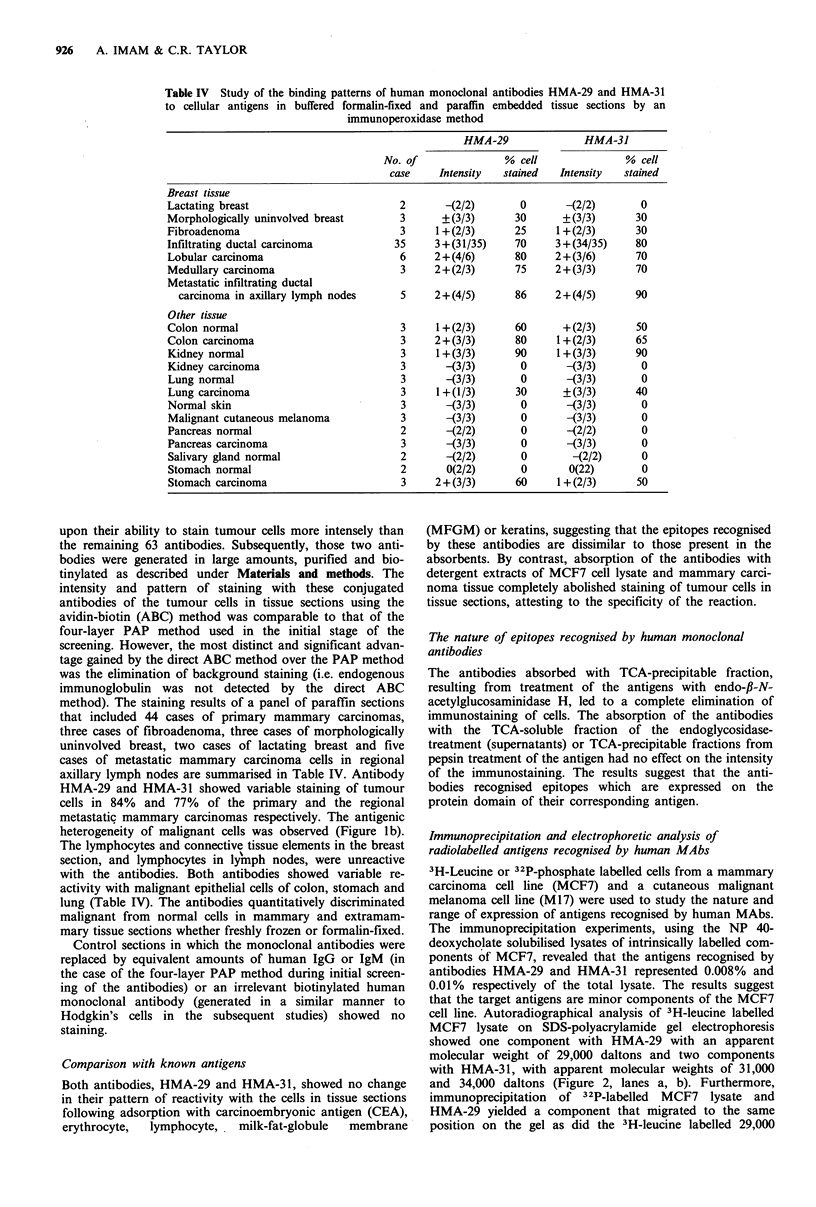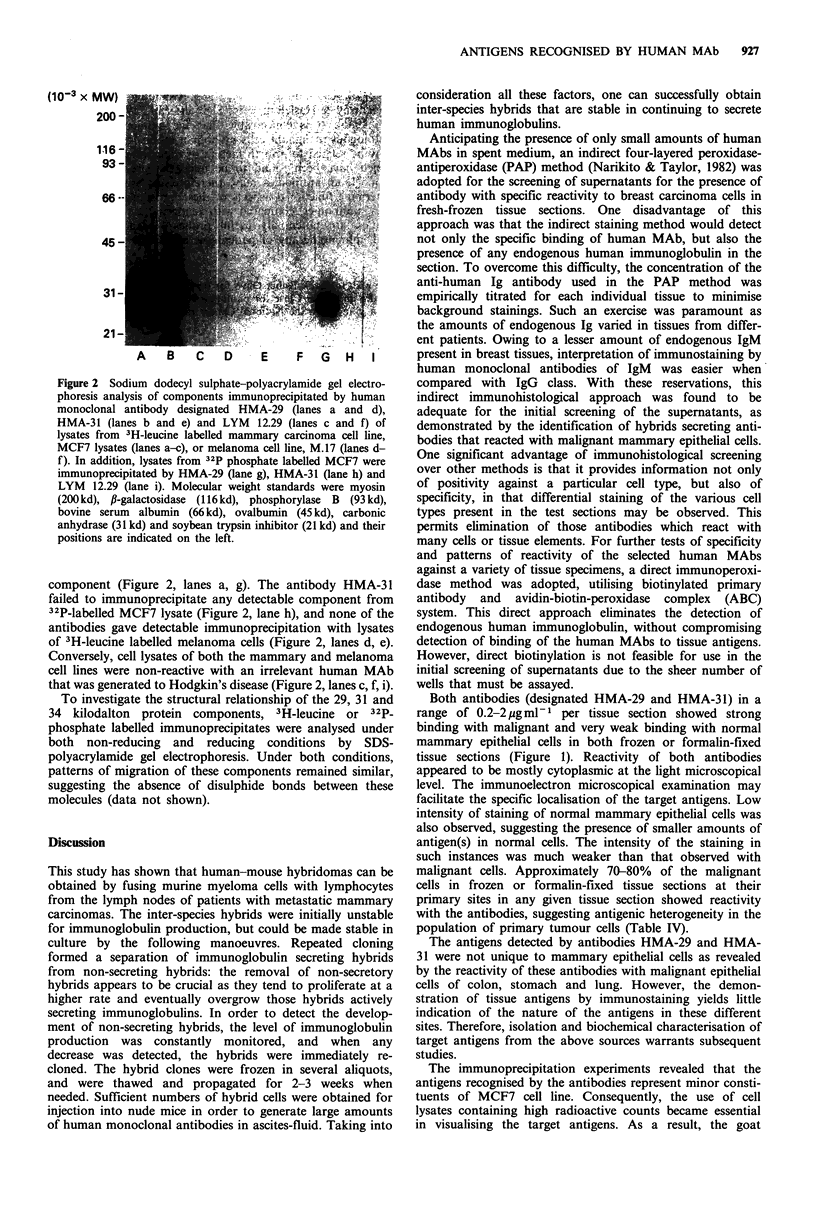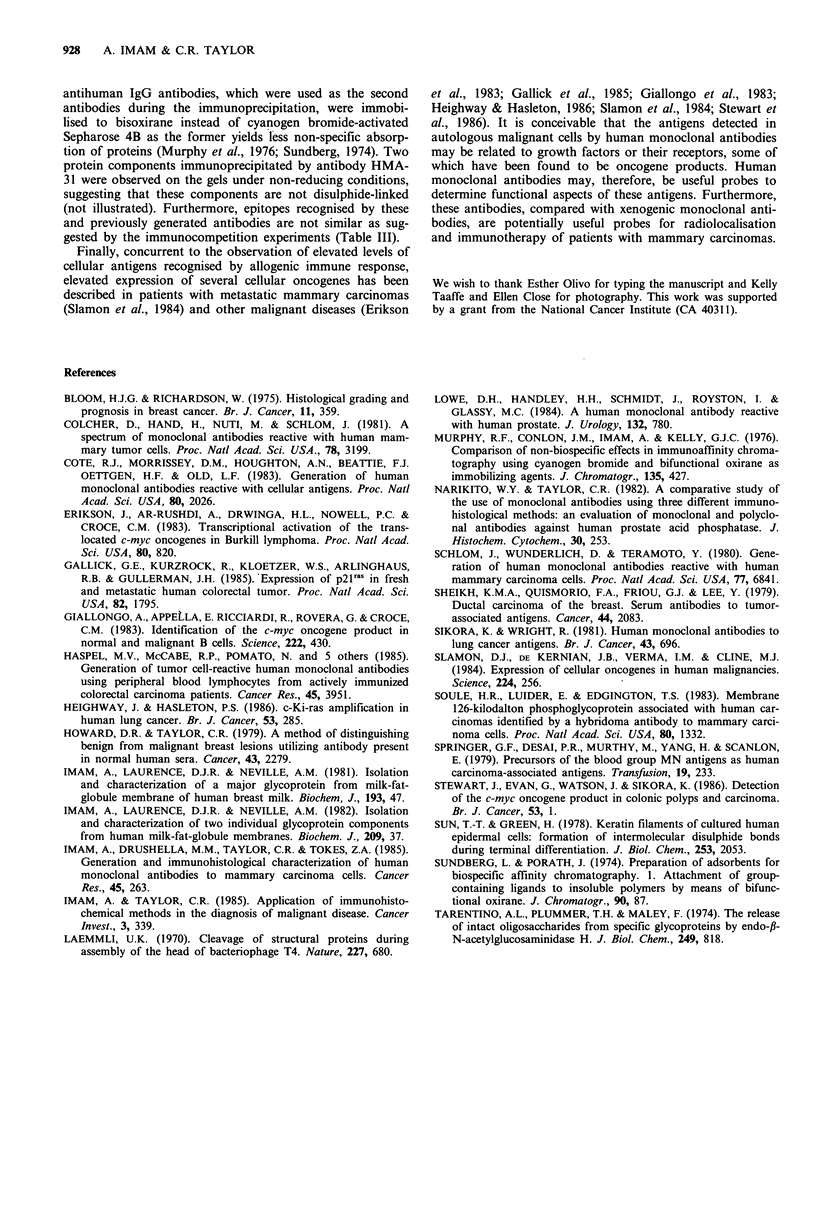# Biochemical and immunological characterizations of antigens recognised by human monoclonal antibodies.

**DOI:** 10.1038/bjc.1989.195

**Published:** 1989-06

**Authors:** A. Imam, C. R. Taylor

**Affiliations:** Department of Pathology, University of Southern California, School of Medicine, Los Angeles 90033.

## Abstract

**Images:**


*Br. J. Cancer* (1989), **59**, 922–928
© The Macmillan Press Ltd., 1989

### Biochemical and immunological characterisations of antigens recognised by human monoclonal antibodies

A. Imam & C.R. Taylor

*Department of Pathology and Comprehensive Cancer Center, University of Southern California, School of Medicine, Los Angeles, CA 90033, USA.*

**Summary** The lymphocytes from lymph nodes of six patients with metastatic mammary carcinomas were hybridised by fusion with a non-secreting variant of murine myeloma cells. Hybrid cells producing human immunoglobulin were detected by screening of culture supernatants using a solid-phase enzyme-linked immunosorbent assay for human IgG or IgM. Reactivity of human immunoglobulins to breast tumour cells was assessed by an indirect immunoperoxidase staining of fresh-frozen breast carcinoma sections. In the initial screening, the tissues used were those removed from the patients who acted as source of lymphocytes for fusion. The hybrid-cells, after repeated cloning, were stable for secretion of immunoglobulins. A total of 14 immunoglobulin G and 51 immunoglobulin M human monoclonal antibodies, showing variable reactivity to mammary carcinoma cells in tissue sections by an indirect immunoperoxidase staining method, were obtained. Two immunoglobulin G monoclonal antibodies (designated HMA-29 and HMA-31) were selected on the basis of their strong reactivity to the tumour cells and utilised to identify their corresponding antigens. The antibodies quantitatively discriminated, as expressed by the degree of staining, malignant from normal or benign mammary epithelia in freshly frozen or formalin-fixed breast tissues. The antibodies also showed reactivity to malignant cells of colon, stomach and lung and to normal cells lining the renal tubules and surface epithelium of colon. As revealed by blocking experiments, the epitopes recognised by these antibodies were not expressed on carcinoembryonic antigens, erythrocytes, lymphocytes, glycoproteins from milk-fat-globule membrane or keratins. The antibody HMA-29 immunoprecipitated a phosphoprotein ($M_r$=29,000), and antibody HMA-31 two protein components ($M_r$=31,000 and 34,000), from lysates of intrinsically labelled human mammary carcinoma cell line (MCF7). Neither of these proteins were present in detectable amounts in an intrinsically labelled melanoma cell line. Immunoblocking and immunoprecipitation experiments suggested that epitopes recognised by these two antibodies are dissimilar and are expressed on different molecules. The antibodies appear to be useful for functional characterisation of those antigens which are present in elevated levels in malignant compared with normal mammary epithelia.

The evidence for the presence of mammary carcinoma-associated antigens has been reported (Howard & Taylor, 1979; Springer *et al.*, 1979; Sheiks *et al.*, 1979). Conversely, antibodies present in the serum of patients with mammary carcinoma have also been shown to be reactive with the carcinoma cells (Colcher *et al.*, 1981; Soule *et al.*, 1983). Accordingly, attempts have been made to utilise lymphocytes from patients with metastatic malignant diseases to produce human monoclonal antibodies by the hybridoma technique (Cote *et al.*, 1983; Haspel *et al.*, 1985; Imam *et al.*, 1985; Low *et al.*, 1984; Schlom *et al.*, 1980; Sikora *et al.*, 1981). In order to generate such human antibodies, lymphocytes were taken from the draining lymph nodes of patients with metastatic mammary carcinoma and fused with non-secretory variant of mouse myeloma cells to obtain human immunoglobulin secreting hybrids.

This paper reports the generation and application of human monoclonal antibodies as probes to identify and characterise antigens which are present at elevated levels in malignant compared with normal mammary epithelia.

### Materials and methods

### *Materials*

Aminopterin, thymidine and hypoxanthine were obtained from Sigma Chemicals (St Louis, MO, USA) and polyethylene glycol 1500 from Aldrich Chemical Co. (Milwaukee, WI, USA). Chromatographically purified human IgM and IgG, rabbit antihuman IgM ($\mu$-chain specific), rabbit antihuman IgG (gamma-chain specific), $F(ab)'_2$ fragment of goat antihuman Fab, rabbit antihuman kappa and lambda light chains, mouse IgG and IgM and sheep antimouse immunoglobulins with no cross reactivity to human immunoglobulins were the products of Cappel Laboratories. Tissue culture reagents were purchased from Flow Laboratories CA, USA) and a gamma horse serum from Bio-Cells Laboratory (Carson, CA, USA).

### *Cell fusion and cloning*

Portions of axillary lymph nodes from patients with metastatic breast carcinoma were obtained and processed as described previously in order to obtain live human lymphocytes in suspension (Imam *et al.*, 1985). The lymphocytes and mouse myeloma cells (M5, a non-secreting and horse serum adapted subline of SP2/OAg 14) were mixed at a ratio of 2.5 to 1, respectively, and fused using 34% (v/v) polyethylene glycol (mol. wt 1,500 daltons) as described previously (Imam *et al.*, 1985). The hybrid cells secreting human immunoglobulin with reactivity to breast carcinoma cells in tissue sections were cloned by limiting dilution. Using this procedure, cloning efficiency varied from 29 to 54% (average 44%).

Spent-media from wells containing hybrid-cells were assayed for the presence of human IgG or IgM by solid phase enzyme-linked immunosorbent assay (ELISA) as described previously (Imam *et al.*, 1985).

### *Production and purification of human monoclonal antibodies*

The human-mouse hybrid clones, designated as HMA-29 ($IgG_1$ antibody), HMA-31 ($IgG_2$ antibody), selected on the basis of production of antibodies with strong reactivity to mammary carcinoma cells in tissue sections, were injected intraperitoneally into Balb/C nude mice which had been primed with pristane 3 weeks earlier. Two to three weeks later, ascites fluid was harvested from the mice and clarified by centrifugation at 12,000$g$ and 4°C for 15 min. The immunoglobulin from ascites fluid was purified as described

Correspondence: Ashraf Imam, Cancer Research Laboratory, U.S.C. School of Medicine, 1303 N. Mission Road, Los Angeles, CA 90033, USA.

previously (Imam *et al.*, 1985). The purified antibodies were conjugated with biotinyl-*N*-hydroxysuccinimide ester as described previously (Imam *et al.*, 1985).

*Preparation of tissue sections*

Uninvolved and malignant human tissues were obtained from the surgical pathology files of the University of Southern California/Los Angeles County Medical Center. Tissues used were either frozen in liquid nitrogen or fixed in 10% buffered-formalin. The fixed paraffin embedded tissue was sectioned at 5 $\mu$m in thickness. Representative tissue-sections were stained with Haematoxylin and Eosin to confirm the diagnosis before immunoperoxidase staining.

*Comparison of epitopes recognised by human MAbs*

To determine whether the presently generated two antibodies and those generated previously, termed CA-27 (25) and JD-39 (22) (Imam *et al.*, 1985), recognise similar or different epitopes, blocking assays using immunohistological techniques were performed as described previously (Imam *et al.*, 1985). The antibodies CA-27 (25) or JD-39 (22) were generated previously in a similar manner. The ability of these antibodies to discriminate malignant from normal cells in breast tissues was not as significant as those presently prepared, HMA-29 and HMA-31.

*Immunocytochemical localisation of tissue antigens*

The four-layer unlabelled antibody peroxidase-antiperoxidase, PAP, and an avidin-biotin-peroxidase complex, ABC, methods were used for localising tissue antigens with the human monoclonal antibodies (Imam & Taylor, 1985).

*PAP method of staining*

This method of staining was employed during the initial period of screening. Human monoclonal antibodies produced by hybrids in tissue-culture-medium were tested for their ability to bind to antigens in histological sections of breast tissue. The tumour tissues used in the primary screening were obtained from the patients who acted as sources of lymphocytes for fusion. Rabbit antihuman IgG or IgM and swine antirabbit antibody were used as the 'link' or 'bridge antibodies' between the human monoclonal antibody (primary) and rabbit peroxidase-antiperoxidase (PAP) complex. Controls included the replacement of primary antibody by an irrelevant human monoclonal antibody to Hodgkin's cells. (Antibody secreted by hybrid that was obtained by fusing mouse myeloma cells with lymphocytes of lymph node from patients with Hodgkin's disease. The antibody showed strong reactivity with Reed-Sternberg cell (manuscipt in preparation).) The tissue sections were incubated with each antibody in an appropriate dilution for 60 min at room temperature. Following each incubation period, the sections were washed with PBS for 15 min. The remainder of the procedures was as described previously (Imam *et al.*, 1985).

*The ABC method of staining*

Biotinylated human monoclonal antibodies (10 $\mu$g ml$^{-1}$) were applied directly to tissue sections. Following the washing of the tissue sections with PBS, avidin-biotin-peroxidase complex in dilution according to vendor's instructions was added. Biotinylated specific antibodies preincubated with an extract of mammary carcinoma cell line, MCF7, or a biotinylated irrelevant human monoclonal antibody to Hodgkin's cells, replacing the same amounts of specific antibody, served as negative controls. Histological classification of mammary epithelial cells was determined according to Bloom & Richardson (1975). The visual estimates of staining intensities were graded as: (−) absent, (±) borderline, (1+) weak, (2+) moderate, (3+) intense. To account for case to case variation in the degree of intensity of staining, any given tumour specimen was evaluated relative to a 'positive control' tissue section containing infiltrating ductal carcinoma cells (and also adjacent normal breast ducts with virtually no reactivity). Visual estimates of the percentage of cells showing reactivity were determined by examining at high magnification (400 ×) five random fields in every tissue section. The mean of counts from the fields examined was recorded as the percentage of cells with staining.

*Absorption of biotinylated human monoclonal antibodies with known antigens*

The biotinylated antibodies, HMA-29 or HMA-31 (1 mg ml$^{-1}$) were separately incubated overnight at 4°C with 10 mg protein preparation from human erythrocytes, lymphocyte, milk-fat-globule membrane (Imam *et al.*, 1981, 1982), keratins (Sun & Green, 1978), the detergent extract of unlabelled MCF7 cell lysates, mammary carcinoma tissue or 1 mg of carcinoembryonic antigen (CEA) immobilised to Sepharose 4B. The procedure of preparing the extract of unlabelled cell lysate was the same as described below for the metabolically labelled cells. Following incubation, the solutions were centrifuged at 100,000 *g* and 4°C for 30 min. The supernatants containing absorbed antibodies were removed and subsequently applied to tissue sections for immunostaining analysis.

*Characterisation of the epitope recognised by human MAbs*

Investigation was conducted to determine whether the human monoclonal antibodies were directed to the protein and/or the carbohydrate portion of antigens recognised by the antibodies as described below.

*Treatment with endo-β-N-acetylglycosaminidase H*

To monitor the cleavage of antigens recognised by HMA-29 or HMA-31 with endo-*β*-*N*-acetylglucosaminidase H, 0.25 $\mu$g protein of MCF7 cell line extract in 100 $\mu$l of 0.1 M sodium citrate buffer, pH 5.5, containing 50 mu of the enzyme were incubated at 37°C for 18 h as described by Tarentino *et al.* (1974). Following incubation, the reaction mixture was mixed with an equal volume of cold 12.5% (w/v) trichloroacetic acid (TCA) for 15 min at 4°C. The mixture was centrifuged at 12,000 *g* and 4°C for 15 min, and the supernatant was removed and dialysed against several changes of PBS at 4°C. The pellet was dissolved in 100 $\mu$l of PBS and dialysed. To ensure a complete precipitation, an appropriate control containing only the antigen in the absence of the enzyme was included.

*Treatment with pepsin*

Two hundred and fifty micrograms of protein from MCF7 cell line extract was dissolved in 100 $\mu$l of 0.07 M sodium acetate buffer, pH 4.0, containing 0.05 M NaCl and 15 $\mu$g of pepsin, and the reaction mixture was incubated at 37°C in a water-bath for 18 h. At the end of the enzymic digestion period, the pH of the solution was adjusted to 8 with 1 N NaOH and was dialysed against several changes of PBS.

*Metabolic labelling of cells and preparation of cell lysate*

Mammary carcinoma cell line, MCF7, and melanoma cell line, M17, were grown as monolayer cultures in 75 mm$^2$ tissue culture flasks and intrinsically labelled when cultures were still subconfluent. The cells were labelled for 24–48 h with either 2 mCi of $^3$H-leucine (110 Ci mmol$^{-1}$) or 10 mCi of $^{32}$P-phosphate (carrier-free) per flask of leucine or phosphate-free DME medium respectively. Following incubation, the cells were washed three times and lysed with 0.05 M Tris-HCl buffer, pH 7.5, containing 0.15 M NaCl, 0.5% (v/v) Nonidet P-40 (NP-40), 0.5% (w/v) sodium deoxycholate, 1 mM phenylmethylsulphonyl fluoride and 0.5 mM chloromethyl-L-(2-phenyl-1-*p*-toluenesulphosnamide) ethyl ketone on ice for 15 min. The lysates were centrifuged at 40,000 *g* and 4°C for 20 min. The supernatants containing detergent-solubilised materials were subsequently used for immunoprecipitation.

*Immunoprecipitation of extracts of radiolabelled cells with human monoclonal antibodies*

The radiolabelled cell lysates (approximately 400 ng of protein containing $5 \times 10^7$ c.p.m.) were mixed with 100 $\mu$l of either a specific human MAb (1.0 mg ml$^{-1}$) or an irrelevant human MAb generated by fusing lymphocytes from patients with Hodgkin's disease. The latter antibody served as a negative control. The mixtures were incubated at 4°C for 16 h. Following the incubation, a 100 $\mu$l suspension of Sepharose 4B conjugated to goat antihuman IgG as described above was added to each reaction mixture. The samples were incubated for a further period of 60 min and centrifuged at 5,000 *g* for 5 min. Following the removal of supernatant by aspiration, the pellet was washed five times with 0.05 M NaCl, 1.0% (w/v) ovalbumin and 0.2% (v/v) NP-40 to remove any non-specifically bound radioactivity. No radioactivity was detectable in the supernatant of the fifth wash.

*SDS-Polyacrylamide gel electrophoresis*

The materials immunoprecipitated with the human MAbs were subsequently analysed by SDS-polyacrylamide gel electrophoresis. The washed pellets were solubilised in 0.05 M Tris-HCl buffer, pH 6.8, containing SDS and 2-mercaptoethanol, boiled for 5 min and centrifuged at 8,000 *g* for 5 min at room temperature. The supernatants were subjected to electrophoresis in 7.5% polyacrylamide slab gels in the presence of SDS by the method of Laemmli (1970). A constant current of 30 mA was applied to each gel for 3–4 h until the dye front approached to within 1 cm of the bottom. The gels were then fixed and stained with 0.25% (w/v) Coomassie blue in a solution containing 50% (v/v) isopropyl alcohol and 10% (v/v) glacial acetic acid. Destaining was performed in a solution containing 10% (v/v) isopropyl alcohol and 10%(v/v) glacial acetic acid. The destained gels were treated with 'Enhance' (New England Nuclear, Boston, MA, USA) and dried on a Whatman no. 3 MM filter paper under reduced pressure, and the radioactive components were visualised by fluorography.

### Results

*Generation and cloning of human-mouse hybrid cells*

Fourteen IgG and 51 IgM producing stable hybridomas were obtained. The hybridomas were obtained by fusing lymphocytes (obtained from the regional lymph nodes of six different patients with metastatic mammary carcinoma) with a non-secretory variant of mouse myeloma cell line (M5, a horse serum-adapted subline of SP2/OAg 14) (Tables I and II).

*Screening assay for the presence of human immunoglobulins*

The enzyme-linked immunoabsorbent assays (ELISA) were employed for the detection of human IgG or IgM secreted by the hybrid-cells in spent media. The specificity and sensitivity of the assays for human IgG or IgM. The sensitivity of the assay was 0.12 $\mu$g ml$^{-1}$ for the detection of IgG or IgM. The assay was specific to its corresponding antigen within the range of 0.03–5.0 $\mu$g ml$^{-1}$ of detection. No cross-reactivity between human and mouse immunoglobulins in this range of detection was observed. Furthermore, human IgG and IgM showed no cross-reactivity in the assay. On average, 32% of the wells containing hybrid-cells were

**Table I** Generation of human-mouse hybrids that secreted human immunoglobulins

| Fusion | No.. of wells seeded | No. of wells with hybrids | No. of wells with hybrids producing human Ig[a] |
|---|---|---|---|
| 1 | 94 | 39 | 19[b] |
| 2 | 243 | 123 | 21[b] |
| 3 | 138 | 61 | 32[b] |
| 4 | 176 | 82 | 27[b] |
| 5 | 45 | 28 | 11[b] |
| 6 | 116 | 57 | 16[b] |
| Total | 812 | 390 (48%) | 126 (32%) |

[a]Number of wells with growing hybrids with >0.5 $\mu$g immunoglobulin per ml of spent medium. [b]Following immunohistological screening for specificity, hydrids from one of each of these wells were selected and cloned (see Table II).

**Table II** Secretion and binding reactivity of human monocloncal antibodies produced by cloned hybrids

| Fusion no.[a] | Wells with single clone after cloning of Ig positive hybrids | No. of hybrid clones with human | | No. of hybrid clones producing antibodies with reactivity to breast tumour cells | |
|---|---|---|---|---|---|
| | | IgG | IgM | IgG | IgM |
| 1 | 97 (45%) | 9 | 21 | 3 | 8 |
| 2 | 56 (29%) | 2 | 17 | 1 | 11 |
| 3 | 86 (45%) | 29 | 6 | 7 | 4 |
| 4 | 71 (37%) | 7 | 8 | 1 | 3 |
| 5 | 89 (46%) | 0 | 23 | – | 16 |
| 6 | 104 (54%) | 5 | 19 | 2 | 9 |
| Total | 503 (44%) | 52 (10%) | 94 (19%) | 14 (27%) | 51 (54%) |

[a]Hybrids from six immunoglobulin positive wells (see Table I and column 4, one from each fusion) were selected, cloned and plated into two 96-well plates. The parameters of selection for cloning of hybrids were based upon their ability to secrete higher amounts of Ig with strong reactivity to tumour cells in tissue sections more than the Ig produced by the remaining hybrids.

obtained from lymphocytes from patients with metastatic breast carcinomas synthesised human immunoglobulins (Table I). Hybrids from six immunoglobulin positive wells, one deriving from each fusion (Table I, column 4), were selected, cloned and plated into two 96-well plates. The parameters of selection for cloning of hybrids were based upon their ability to secrete higher amounts of Ig with strong reactivity to tumour in comparison with normal mammary epithelial cells in frozen tissue sections. An indirect immunoperoxidase technique of screening for Ig binding, as described in the **Methods** section, was employed. Fifty-two IgG and 94 IgM monoclonal antibodies producing clones were obtained (Table II). Of these antibodies, 14 IgG (27%) and 51 IgM (54%) showed evidence of reactivity to tumour cells as described above (Table II). The levels of human immunoglobulin produced by cloned hybrids varied from 0.5 to 2.0 $\mu$g of IgG or 2.5 to 5.0 $\mu$g of IgM per ml of spent medium. The human MAbs in ascites yielded human IgG or IgM within a range of 2–4 mg ml$^{-1}$. For accurate determination of the concentration of affinity purified IgG monoclonal antibodies, their corresponding purified isotypes of human IgG (i.e. $IgG_1$, $IgG_2$, $IgG_3$, $IgG_4$) were used as standards in ELISA.

The majority of interspecies hybrids were not stable for producing human immunoglobulins. However, an early cloning (post-fusion days 25–30), and repeated cloning (post-cloning days 40–60 and 110–120), of the positive hybrids appears to enhance their stability in continuing to produce immunoglobulins.

**Table III** Determination of epitopes recognised by human monoclonal antibodies by an immunohistological technique

| *Incubated with* | *Wash with* | *Incubated with* | *Wash with* | *Incubated with* | *Wash with* | *Incubated with* | *Intensity of staining of mammary carcinoma cells*[c] |
|---|---|---|---|---|---|---|---|
| PBS | PBS | HMA-29[d] | PBS | ABC[a] | PBS | AEC-$H_2O_2$[b] | 3+ |
| HMA-29[e] | PBS | HMA-29[d] | PBS | ABC[a] | PBS | AEC-$H_2O_2$[b] | – |
| HMA-31[e] | PBS | HMA-29[d] | PBS | ABC[a] | PBS | AEC-$H_2O_2$[b] | 3+ |
| PBS | PBS | HMA-31[d] | PBS | ABC[a] | PBS | AEC-$H_2O_2$[b] | 3+ |
| HMA-31[e] | PBS | HMA-31[d] | PBS | ABC[a] | PBS | AEC-$H_2O_2$[b] | – |
| HMA-29[e] | PBS | HMA-31[d] | PBS | ABC[a] | PBS | AEC-$H_2O_2$[b] | 3+ |
| PBS | PBS | CA27(25)[d,f] | PBS | ABC[a] | PBS | AEC-$H_2O_2$[b] | 3+ |
| CA27(25)[e,f] | PBS | CA27(25)[d,f] | PBS | ABC[a] | PBS | AEC-$H_2O_2$[b] | – |
| HMA-29[e] | PBS | CA27(25)[d,f] | PBS | ABC[a] | PBS | AEC-$H_2O_2$[b] | 3+ |
| HMA-31[e] | PBS | CA27(25)[d,f] | PBS | ABC[a] | PBS | AEC-$H_2O_2$[b] | 3+ |
| PBS | PBS | JD39(22)[d,f] | PBS | ABC[a] | PBS | AEC-$H_2O_2$[b] | 3+ |
| JD39(22)[e,f] | PBS | JD39(22)[d,f] | PBS | ABC[a] | PBS | AEC-$H_2O_2$[b] | – |
| HMA-29[e] | PBS | JD39(22)[d,f] | PBS | ABC[a] | PBS | AEC-$H_2O_2$[b] | 3+ |
| HMA-31[e] | PBS | JD39(22)[d,f] | PBS | ABC[a] | PBS | AEC-$H_2O_2$[b] | 3+ |

[a]Avidin-biotin-peroxidase complex (ABC); [b]aminoethyl carbazole-hydrogen peroxide; [c]absence of staining – intense staining 3+; [d]biotinylated human monoclonal antibody; [e]unlabelled human monoclonal antibody; [f]Imam *et al.* (1985).

*Comparison of epitopes recognised by human monoclonal antibodies*

The experiments were performed to determine any similarity between epitopes recognised by presently generated human monoclonal antibodies (HMA-29, HMA-31) and previously generated antibodies (CA-27 (25) or JD-39 (22)) (Imam *et al.*, 1985). The immunoblocking experiments showed that the antigenic binding sites for antibody HMA-29 were not blocked by antibody HMA-31. Conversely, the reactivity of HMA-31 was not obstructed by antibody HMA-29 (Table III). Indeed, the antigens recognised by these antibodies are also different with respect to their molecular weights (Figure 2, lanes a, b). Furthermore, the binding activity of HMA-29 and HMA-31 was not blocked by any of the previously generated antibodies CA-27 (25) or JD-39 (22) (Table III). The results indicate that two different epitopes are recognised by the antibodies and are not related to epitopes recognised by previously generated antibodies.

*Localisation of cellular antigens with human monoclonal antibodies*

Spent medium from each of the immunoglobulin-producing clones was initially screened for binding activity to tissue sections of autologous breast carcinoma using an indirect immunoperoxidase (PAP) staining technique. The indirect four-layer PAP methods gave some background staining, attributable to detection of endogenous IgM or IgG in breast tissues. Endogenous immunoglobulin sensitivity was more notable while staining for human monoclonal antibodies of IgG class, but did not interefere with the interpretation of staining patterns, which in all cases were assessed with reference to positive and negative controls as described in the methods section. The findings are summarised in Table II (columns 5, 6).

Fourteen IgG and 51 IgM human MAbs showed staining of tumour cells with a variable intensity in all cases of infiltrating ductal carcinoma of the breast (Table II). Under these conditions, lymphocytes, blood vessels and stromal elements failed to stain. Weak staining of morphologically normal breast epithelial cells present in the same tissue section was observed in some cases. However, the intensity of staining in such cases was much less than for malignant cells (Figure 1a).

Of these 65 MAbs, two antibodies designated HMA-29 ($IgG_1$ antibody) and HMA-31 ($IgG_2$ antibody), were selected for further study. The chief parameter for selection rested

**Figure 1** Binding pattern of a human monoclonal antibody HMA-29 to mammary epithelial cells in formalin-fixed and paraffin-embedded tissue sections by a direct immunoperoxidase (avidin-biotin-peroxidase) method. The biotinylated human monoclonal antibody was applied at a concentration of $10\,\mu g\,ml^{-1}$. The sections were counterstained with Mayer's haematoxylin. The stromal components were consistently negative. (**a**) Normal and infiltrating ductal carcinoma of breast. The uninvolved duct at the lower left side (short arrow) is virtually unstained whereas surrounding malignant cells (long arrow) showed strong reactivity of cytoplasmic components with the antibody (original mag. ×150). (**b**) Infiltrating ductal carcinoma. Not all tumour cells in certain cases showed reactivity (absence of staining is indicated by short arrow), indicating antigenic heterogeneity among the tumour cell population (original mag. ×312).

**Table IV** Study of the binding patterns of human monoclonal antibodies HMA-29 and HMA-31 to cellular antigens in buffered formalin-fixed and paraffin embedded tissue sections by an immunoperoxidase method

| | | *HMA-29* | | *HMA-31* | |
|---|---|---|---|---|---|
| | *No. of case* | *Intensity* | *% cell stained* | *Intensity* | *% cell stained* |
| *Breast tissue* | | | | | |
| Lactating breast | 2 | –(2/2) | 0 | –(2/2) | 0 |
| Morphologically uninvolved breast | 3 | ±(3/3) | 30 | ±(3/3) | 30 |
| Fibroadenoma | 3 | 1+(2/3) | 25 | 1+(2/3) | 30 |
| Infiltrating ductal carcinoma | 35 | 3+(31/35) | 70 | 3+(34/35) | 80 |
| Lobular carcinoma | 6 | 2+(4/6) | 80 | 2+(3/6) | 70 |
| Medullary carcinoma | 3 | 2+(2/3) | 75 | 2+(3/3) | 70 |
| Metastatic infiltrating ductal carcinoma in axillary lymph nodes | 5 | 2+(4/5) | 86 | 2+(4/5) | 90 |
| *Other tissue* | | | | | |
| Colon normal | 3 | 1+(2/3) | 60 | +(2/3) | 50 |
| Colon carcinoma | 3 | 2+(3/3) | 80 | 1+(2/3) | 65 |
| Kidney normal | 3 | 1+(3/3) | 90 | 1+(3/3) | 90 |
| Kidney carcinoma | 3 | –(3/3) | 0 | –(3/3) | 0 |
| Lung normal | 3 | –(3/3) | 0 | –(3/3) | 0 |
| Lung carcinoma | 3 | 1+(1/3) | 30 | ±(3/3) | 40 |
| Normal skin | 3 | –(3/3) | 0 | –(3/3) | 0 |
| Malignant cutaneous melanoma | 3 | –(3/3) | 0 | –(3/3) | 0 |
| Pancreas normal | 2 | –(2/2) | 0 | –(2/2) | 0 |
| Pancreas carcinoma | 3 | –(3/3) | 0 | –(3/3) | 0 |
| Salivary gland normal | 2 | –(2/2) | 0 | –(2/2) | 0 |
| Stomach normal | 2 | 0(2/2) | 0 | 0(22) | 0 |
| Stomach carcinoma | 3 | 2+(3/3) | 60 | 1+(2/3) | 50 |

upon their ability to stain tumour cells more intensely than the remaining 63 antibodies. Subsequently, those two antibodies were generated in large amounts, purified and biotinylated as described under **Materials and methods**. The intensity and pattern of staining with these conjugated antibodies of the tumour cells in tissue sections using the avidin-biotin (ABC) method was comparable to that of the four-layer PAP method used in the initial stage of the screening. However, the most distinct and significant advantage gained by the direct ABC method over the PAP method was the elimination of background staining (i.e. endogenous immunoglobulin was not detected by the direct ABC method). The staining results of a panel of paraffin sections that included 44 cases of primary mammary carcinomas, three cases of fibroadenoma, three cases of morphologically uninvolved breast, two cases of lactating breast and five cases of metastatic mammary carcinoma cells in regional axillary lymph nodes are summarised in Table IV. Antibody HMA-29 and HMA-31 showed variable staining of tumour cells in 84% and 77% of the primary and the regional metastatic mammary carcinomas respectively. The antigenic heterogeneity of malignant cells was observed (Figure 1b). The lymphocytes and connective tissue elements in the breast section, and lymphocytes in lymph nodes, were unreactive with the antibodies. Both antibodies showed variable reactivity with malignant epithelial cells of colon, stomach and lung (Table IV). The antibodies quantitatively discriminated malignant from normal cells in mammary and extramammary tissue sections whether freshly frozen or formalin-fixed.

Control sections in which the monoclonal antibodies were replaced by equivalent amounts of human IgG or IgM (in the case of the four-layer PAP method during initial screening of the antibodies) or an irrelevant biotinylated human monoclonal antibody (generated in a similar manner to Hodgkin's cells in the subsequent studies) showed no staining.

### *Comparison with known antigens*

Both antibodies, HMA-29 and HMA-31, showed no change in their pattern of reactivity with the cells in tissue sections following adsorption with carcinoembryonic antigen (CEA), erythrocyte, lymphocyte, milk-fat-globule membrane (MFGM) or keratins, suggesting that the epitopes recognised by these antibodies are dissimilar to those present in the absorbents. By contrast, absorption of the antibodies with detergent extracts of MCF7 cell lysate and mammary carcinoma tissue completely abolished staining of tumour cells in tissue sections, attesting to the specificity of the reaction.

### *The nature of epitopes recognised by human monoclonal antibodies*

The antibodies absorbed with TCA-precipitable fraction, resulting from treatment of the antigens with endo-$\beta$-*N*-acetylglucosaminidase H, led to a complete elimination of immunostaining of cells. The absorption of the antibodies with the TCA-soluble fraction of the endoglycosidase-treatment (supernatants) or TCA-precipitable fractions from pepsin treatment of the antigen had no effect on the intensity of the immunostaining. The results suggest that the antibodies recognised epitopes which are expressed on the protein domain of their corresponding antigen.

### *Immunoprecipitation and electrophoretic analysis of radiolabelled antigens recognised by human MAbs*

$^{3}$H-Leucine or $^{32}$P-phosphate labelled cells from a mammary carcinoma cell line (MCF7) and a cutaneous malignant melanoma cell line (M17) were used to study the nature and range of expression of antigens recognised by human MAbs. The immunoprecipitation experiments, using the NP 40-deoxycholate solubilised lysates of intrinsically labelled components of MCF7, revealed that the antigens recognised by antibodies HMA-29 and HMA-31 represented 0.008% and 0.01% respectively of the total lysate. The results suggest that the target antigens are minor components of the MCF7 cell line. Autoradiographical analysis of $^{3}$H-leucine labelled MCF7 lysate on SDS-polyacrylamide gel electrophoresis showed one component with HMA-29 with an apparent molecular weight of 29,000 daltons and two components with HMA-31, with apparent molecular weights of 31,000 and 34,000 daltons (Figure 2, lanes a, b). Furthermore, immunoprecipitation of $^{32}$P-labelled MCF7 lysate and HMA-29 yielded a component that migrated to the same position on the gel as did the $^{3}$H-leucine labelled 29,000

**Figure 2** Sodium dodecyl sulphate–polyacrylamide gel electrophoresis analysis of components immunoprecipitated by human monoclonal antibody designated HMA-29 (lanes a and d), HMA-31 (lanes b and e) and LYM 12.29 (lanes c and f) of lysates from $^{3}$H-leucine labelled mammary carcinoma cell line, MCF7 lysates (lanes a–c), or melanoma cell line, M.17 (lanes d–f). In addition, lysates from $^{32}$P phosphate labelled MCF7 were immunoprecipitated by HMA-29 (lane g), HMA-31 (lane h) and LYM 12.29 (lane i). Molecular weight standards were myosin (200 kd), $\beta$-galactosidase (116 kd), phosphorylase B (93 kd), bovine serum albumin (66 kd), ovalbumin (45 kd), carbonic anhydrase (31 kd) and soybean trypsin inhibitor (21 kd) and their positions are indicated on the left.

component (Figure 2, lanes a, g). The antibody HMA-31 failed to immunoprecipitate any detectable component from $^{32}$P-labelled MCF7 lysate (Figure 2, lane h), and none of the antibodies gave detectable immunoprecipitation with lysates of $^{3}$H-leucine labelled melanoma cells (Figure 2, lanes d, e). Conversely, cell lysates of both the mammary and melanoma cell lines were non-reactive with an irrelevant human MAb that was generated to Hodgkin's disease (Figure 2, lanes c, f, i).

To investigate the structural relationship of the 29, 31 and 34 kilodalton protein components, $^{3}$H-leucine or $^{32}$P-phosphate labelled immunoprecipitates were analysed under both non-reducing and reducing conditions by SDS-polyacrylamide gel electrophoresis. Under both conditions, patterns of migration of these components remained similar, suggesting the absence of disulphide bonds between these molecules (data not shown).

### Discussion

This study has shown that human–mouse hybridomas can be obtained by fusing murine myeloma cells with lymphocytes from the lymph nodes of patients with metastatic mammary carcinomas. The inter-species hybrids were initially unstable for immunoglobulin production, but could be made stable in culture by the following manoeuvres. Repeated cloning formed a separation of immunoglobulin secreting hybrids from non-secreting hybrids: the removal of non-secretory hybrids appears to be crucial as they tend to proliferate at a higher rate and eventually overgrow those hybrids actively secreting immunoglobulins. In order to detect the development of non-secreting hybrids, the level of immunoglobulin production was constantly monitored, and when any decrease was detected, the hybrids were immediately recloned. The hybrid clones were frozen in several aliquots, and were thawed and propagated for 2–3 weeks when needed. Sufficient numbers of hybrid cells were obtained for injection into nude mice in order to generate large amounts of human monoclonal antibodies in ascites-fluid. Taking into consideration all these factors, one can successfully obtain inter-species hybrids that are stable in continuing to secrete human immunoglobulins.

Anticipating the presence of only small amounts of human MAbs in spent medium, an indirect four-layered peroxidase-antiperoxidase (PAP) method (Narikito & Taylor, 1982) was adopted for the screening of supernatants for the presence of antibody with specific reactivity to breast carcinoma cells in fresh-frozen tissue sections. One disadvantage of this approach was that the indirect staining method would detect not only the specific binding of human MAb, but also the presence of any endogenous human immunoglobulin in the section. To overcome this difficulty, the concentration of the anti-human Ig antibody used in the PAP method was empirically titrated for each individual tissue to minimise background stainings. Such an exercise was paramount as the amounts of endogenous Ig varied in tissues from different patients. Owing to a lesser amount of endogenous IgM present in breast tissues, interpretation of immunostaining by human monoclonal antibodies of IgM was easier when compared with IgG class. With these reservations, this indirect immunohistological approach was found to be adequate for the initial screening of the supernatants, as demonstrated by the identification of hybrids secreting antibodies that reacted with malignant mammary epithelial cells. One significant advantage of immunohistological screening over other methods is that it provides information not only of positivity against a particular cell type, but also of specificity, in that differential staining of the various cell types present in the test sections may be observed. This permits elimination of those antibodies which react with many cells or tissue elements. For further tests of specificity and patterns of reactivity of the selected human MAbs against a variety of tissue specimens, a direct immunoperoxidase method was adopted, utilising biotinylated primary antibody and avidin-biotin-peroxidase complex (ABC) system. This direct approach eliminates the detection of endogenous human immunoglobulin, without compromising detection of binding of the human MAbs to tissue antigens. However, direct biotinylation is not feasible for use in the initial screening of supernatants due to the sheer number of wells that must be assayed.

Both antibodies (designated HMA-29 and HMA-31) in a range of 0.2–2 $\mu$g ml$^{-1}$ per tissue section showed strong binding with malignant and very weak binding with normal mammary epithelial cells in both frozen or formalin-fixed tissue sections (Figure 1). Reactivity of both antibodies appeared to be mostly cytoplasmic at the light microscopical level. The immunoelectron microscopical examination may facilitate the specific localisation of the target antigens. Low intensity of staining of normal mammary epithelial cells was also observed, suggesting the presence of smaller amounts of antigen(s) in normal cells. The intensity of the staining in such instances was much weaker than that observed with malignant cells. Approximately 70–80% of the malignant cells in frozen or formalin-fixed tissue sections at their primary sites in any given tissue section showed reactivity with the antibodies, suggesting antigenic heterogeneity in the population of primary tumour cells (Table IV).

The antigens detected by antibodies HMA-29 and HMA-31 were not unique to mammary epithelial cells as revealed by the reactivity of these antibodies with malignant epithelial cells of colon, stomach and lung. However, the demonstration of tissue antigens by immunostaining yields little indication of the nature of the antigens in these different sites. Therefore, isolation and biochemical characterisation of target antigens from the above sources warrants subsequent studies.

The immunoprecipitation experiments revealed that the antigens recognised by the antibodies represent minor constituents of MCF7 cell line. Consequently, the use of cell lysates containing high radioactive counts became essential in visualising the target antigens. As a result, the goat antihuman IgG antibodies, which were used as the second antibodies during the immunoprecipitation, were immobilised to bisoxirane instead of cyanogen bromide-activated Sepharose 4B as the former yields less non-specific absorption of proteins (Murphy *et al.*, 1976; Sundberg, 1974). Two protein components immunoprecipitated by antibody HMA-31 were observed on the gels under non-reducing conditions, suggesting that these components are not disulphide-linked (not illustrated). Furthermore, epitopes recognised by these and previously generated antibodies are not similar as suggested by the immunocompetition experiments (Table III).

Finally, concurrent to the observation of elevated levels of cellular antigens recognised by allogenic immune response, elevated expression of several cellular oncogenes has been described in patients with metastatic mammary carcinomas (Slamon *et al.*, 1984) and other malignant diseases (Erikson *et al.*, 1983; Gallick *et al.*, 1985; Giallongo *et al.*, 1983; Heighway & Hasleton, 1986; Slamon *et al.*, 1984; Stewart *et al.*, 1986). It is conceivable that the antigens detected in autologous malignant cells by human monoclonal antibodies may be related to growth factors or their receptors, some of which have been found to be oncogene products. Human monoclonal antibodies may, therefore, be useful probes to determine functional aspects of these antigens. Furthermore, these antibodies, compared with xenogenic monoclonal antibodies, are potentially useful probes for radiolocalisation and immunotherapy of patients with mammary carcinomas.

We wish to thank Esther Olivo for typing the manuscript and Kelly Taaffe and Ellen Close for photography. This work was supported by a grant from the National Cancer Institute (CA 40311).

### References

BLOOM, H.J.G. & RICHARDSON, W. (1975). Histological grading and prognosis in breast cancer. *Br. J. Cancer*, **11**, 359.

COLCHER, D., HAND, H., NUTI, M. & SCHLOM, J. (1981). A spectrum of monoclonal antibodies reactive with human mammary tumor cells. *Proc. Natl Acad. Sci. USA.*, **78**, 3199.

COTE, R.J., MORRISSEY, D.M., HOUGHTON, A.N., BEATTIE, F.J. OETTGEN, H.F. & OLD, L.F. (1983). Generation of human monoclonal antibodies reactive with cellular antigens. *Proc. Natl Acad. Sci. USA*, **80**, 2026.

ERIKSON, J., AR-RUSHDI, A., DRWINGA, H.L., NOWELL, P.C. & CROCE, C.M. (1983). Transcriptional activation of the translocated *c-myc* oncogenes in Burkill lymphoma. *Proc. Natl Acad. Sci. USA*, **80**, 820.

GALLICK, G.E., KURZROCK, R., KLOETZER, W.S., ARLINGHAUS, R.B. & GULLERMAN, J.H. (1985). Expression of $p21^{ras}$ in fresh and metastatic human colorectal tumor. *Proc. Natl Acad. Sci. USA*, **82**, 1795.

GIALLONGO, A., APPELLA, E. RICCIARDI, R., ROVERA, G. & CROCE, C.M. (1983). Identification of the *c-myc* oncogene product in normal and malignant B cells. *Science*, **222**, 430.

HASPEL, M.V., McCABE, R.P., POMATO, N. and 5 others (1985). Generation of tumor cell-reactive human monoclonal antibodies using peripheral blood lymphocytes from actively immunized colorectal carcinoma patients. *Cancer Res.*, **45**, 3951.

HEIGHWAY, J. & HASLETON, P.S. (1986). c-Ki-ras amplification in human lung cancer. *Br. J. Cancer*, **53**, 285.

HOWARD, D.R. & TAYLOR, C.R. (1979). A method of distinguishing benign from malignant breast lesions utilizing antibody present in normal human sera. *Cancer*, **43**, 2279.

IMAM, A., LAURENCE, D.J.R. & NEVILLE, A.M. (1981). Isolation and characterization of a major glycoprotein from milk-fat-globule membrane of human breast milk. *Biochem, J.*, **193**, 47.

IMAM, A., LAURENCE, D.J.R. & NEVILLE, A.M. (1982). Isolation and characterization of two individual glycoprotein components from human milk-fat-globule membranes. *Biochem. J.*, **209**, 37.

IMAM, A., DRUSHELLA, M.M., TAYLOR, C.R. & TOKES, Z.A. (1985). Generation and immunohistological characterization of human monoclonal antibodies to mammary carcinoma cells. *Cancer Res.*, **45**, 263.

IMAM, A. & TAYLOR, C.R. (1985). Application of immunohistochemical methods in the diagnosis of malignant disease. *Cancer Invest.*, **3**, 339.

LAEMMLI, U.K. (1970). Cleavage of structural proteins during assembly of the head of bacteriophage T4. *Nature*, **227**, 680.

LOWE, D.H., HANDLEY, H.H., SCHMIDT, J., ROYSTON, I. & GLASSY, M.C. (1984). A human monoclonal antibody reactive with human prostate. *J. Urology*, **132**, 780.

MURPHY, R.F., CONLON, J.M., IMAM, A. & KELLY, G.J.C. (1976). Comparison of non-biospecific effects in immunoaffinity chromatography using cyanogen bromide and bifunctional oxirane as immobilizing agents. *J. Chromatogr.*, **135**, 427.

NARIKITO, W.Y. & TAYLOR, C.R. (1982). A comparative study of the use of monoclonal antibodies using three different immunohistological methods: an evaluation of monoclonal and polyclonal antibodies against human prostate acid phosphatase. *J. Histochem. Cytochem.*, **30**, 253.

SCHLOM, J., WUNDERLICH, D. & TERAMOTO, Y. (1980). Generation of human monoclonal antibodies reactive with human mammary carcinoma cells. *Proc. Natl Acad. Sci. USA*, **77**, 6841.

SHEIKH, K.M.A., QUISMORIO, F.A., FRIOU, G.J. & LEE, Y. (1979). Ductal carcinoma of the breast. Serum antibodies to tumor-associated antigens. *Cancer*, **44**, 2083.

SIKORA, K. & WRIGHT, R. (1981). Human monoclonal antibodies to lung cancer antigens. *Br. J. Cancer*, **43**, 696.

SLAMON, D.J., DE KERNIAN, J.B., VERMA, I.M. & CLINE, M.J. (1984). Expression of cellular oncogenes in human malignancies. *Science*, **224**, 256.

SOULE, H.R., LUIDER, E. & EDGINGTON, T.S. (1983). Membrane 126-kilodalton phosphoglycoprotein associated with human carcinomas identified by a hybridoma antibody to mammary carcinoma cells. *Proc. Natl Acad. Sci. USA*, **80**, 1332.

SPRINGER, G.F., DESAI, P.R., MURTHY, M., YANG, H. & SCANLON, E. (1979). Precursors of the blood group MN antigens as human carcinoma-associated antigens. *Transfusion*, **19**, 233.

STEWART, J., EVAN, G., WATSON, J. & SIKORA, K. (1986). Detection of the *c-myc* oncogene product in colonic polyps and carcinoma. *Br. J. Cancer*, **53**, 1.

SUN, T.-T. & GREEN, H. (1978). Keratin filaments of cultured human epidermal cells: formation of intermolecular disulphide bonds during terminal differentiation. *J. Biol. Chem.*, **253**, 2053.

SUNDBERG, L. & PORATH, J. (1974). Preparation of adsorbents for biospecific affinity chromatography. 1. Attachment of group-containing ligands to insoluble polymers by means of bifunctional oxirane. *J. Chromatogr.*, **90**, 87.

TARENTINO, A.L., PLUMMER, T.H. & MALEY, F. (1974). The release of intact oligosaccharides from specific glycoproteins by endo-$\beta$-N-acetylglucosaminidase H. *J. Biol. Chem.*, **249**, 818.